# Physiological and Ultrastructural Responses to Excessive-Copper-Induced Toxicity in Two Differentially Copper Tolerant Citrus Species

**DOI:** 10.3390/plants12020351

**Published:** 2023-01-11

**Authors:** Xin-Yu Li, Mei-Lan Lin, Fei Lu, Xin Zhou, Xing Xiong, Li-Song Chen, Zeng-Rong Huang

**Affiliations:** 1 College of Resources and Environment, Fujian Agriculture and Forestry University, Fuzhou 350002, China; 2 Key Lab of Soil Ecosystem Health and Regulation, Fujian Province University (Fujian Agriculture and Forestry University), Fuzhou 350002, China

**Keywords:** anatomy, *Citrus grandis*, *Citrus sinensis*, copper toxicity, ultrastructure

## Abstract

Over-applied copper (Cu)-based agrochemicals are toxic to citrus trees. However, less information is available discussing the ultrastructural alterations in Cu-stressed citrus species. In the present study, seedlings of *Citrus sinensis* and *Citrus grandis* that differed in Cu-tolerance were sandy-cultured with nutrient solution containing 0.5 µM Cu (as control) or 300 µM Cu (as Cu toxicity) for 18 weeks. At the end of the treatments, the physiological parameters and ultrastructural features of the citrus leaves and roots were analyzed. The results indicate that Cu toxicity significantly decreased the ratio of shoot biomass to dry weight, the Cu translocation factor and the total chlorophyll of two citrus species. The anatomical and ultrastructural alterations verified that excessive Cu resulted in starch granules accumulated in the leaves and roots of the two citrus species. Under Cu toxicity, increased root flocculent precipitate and thickened root cell wall might reduce the Cu translocation from citrus roots to the shoots. Compared with *C. sinensis*, *C. grandis* maintained a relatively integral root cellular structure under Cu toxicity, which provided a structural basis for a higher Cu tolerance than *C. sinensis*. The present results increase our understanding of the physiological and ultrastructural responses to Cu toxicity in citrus species.

## 1. Introduction

The soil copper (Cu) contamination induced by the over-application of Cu-based agrochemicals is a primary environmental and toxicological concern for sustainable agriculture [1,2]. In citrus orchard management, Cu-plus fungicides (such as Bordeaux mixture) and micronutrient fertilizers are frequently foliar-sprayed for pathogen controlling and nutrient adjustment, respectively [3,4]. The Cu-containing solution dripped from the trees or washed by precipitation increased the Cu concentration of the topsoil. Accordingly, old citrus orchards often had a significantly higher soil Cu concentration than younger ones [5,6]. Similar results were also reported in vineyards soils [7] and apple orchards [8].

It has been generally accepted that Cu concentrations (extracted with DPTA) above 5.0 μg/g [9] and 20.0 μg/g [10,11] represent excessive Cu levels in citrus orchard soil and citrus leaves, respectively. According to our previous investigation of the mineral nutrients in pummelo orchards in the Fujian province of China (309 samples), 28.3% of tested soils and 70.0% of pummelo leaves had excessive Cu [12], which induced potential Cu toxicity in citrus. Excessive Cu absorption by citrus roots resulted in oxidative stress, which inhibited root elongation and decreased the number of root branches [13]. Citrus leaves suffering from Cu toxicity are yellowish with lower chlorophyll content and photosynthetic rate [14]. Moreover, Cu phytotoxicity decreased the fruiting number of citrus species [15] and caused visible damage on the fruit surface, which reduced its profitability markedly.

Revealing the Cu-tolerant mechanisms of citrus species is crucial for optimized Cu-nutrient management and for improving environmental quality in the sustainable development of citrus production. For those purposes, we have investigated the Cu tolerance of citrus seedlings at the biochemical and physiological [16], transcriptional [17], proteomic [18] and metabolomic levels [19]. Our previous findings indicate that activated antioxidative defenses [19] promote Cu retention by the root cell wall [20] and secretion of root exudates [21] represented the most critical responses to Cu toxicity in citrus species. Despite versatile Cu-tolerant mechanisms of a biochemical or molecular basis being unveiled, evidence regarding ultrastructural alterations of citrus species under Cu toxicity is still missing.

The alterations of plant tissues and organs provide visible and direct evidence for a better understanding of plant physiological responses to Cu toxicity. For instance, Sánchez-Pardo et al. [22] revealed that a higher Cu tolerance of soybean than white lupin was associated with the size of palisade parenchyma and epidermal cells, which accounted for the Cu micro localization. Likewise, de Freitas et al. [23] have reported that Cu toxicity caused membrane and endoderm ruptures, inhibiting the root nutrient uptake and translocation from roots to shoots in *Inga subnuda* based on anatomical and ultrastructural analyses. Furthermore, using TEM, Minkina et al. [24] revealed that Cu toxicity disorganized the thylakoid membranes of leaves and damaged the integrity of the root cell wall and cytoplasmic membrane in barley (*Hordeum sativum*). The existing studies identified that the ultrastructural alteration of plant tissues exposing Cu toxicity varied with plant species and Cu tolerance. Therefore, the anatomical and ultrastructural investigations would offer vital insights into the Cu tolerance of citrus species.

The present study aimed to explore the anatomical and ultrastructural alterations in the leaves and roots of *C. sinensis* and *C. grandis* that were subjected to 300 µM Cu toxicity for 18 weeks. At the end of treatments, the physiological parameters, including biomass distribution, the Cu translocation and leaf total chlorophyll content, were measured. The anatomical features of citrus leaves and roots were examined under SEM. Additionally, the ultrastructural characteristics of citrus leaves and roots were observed by TEM. The results of the present study would extend our understanding of the physiological responses of citrus species conferring Cu toxicity on an ultrastructural basis. 

## 2. Results

### 2.1. Cu Toxicity Downregulated Seedling Height, Induced Leaf Chlorosis and Hampered Root Development of C. sinensis and C. grandis

**Figure 1:**
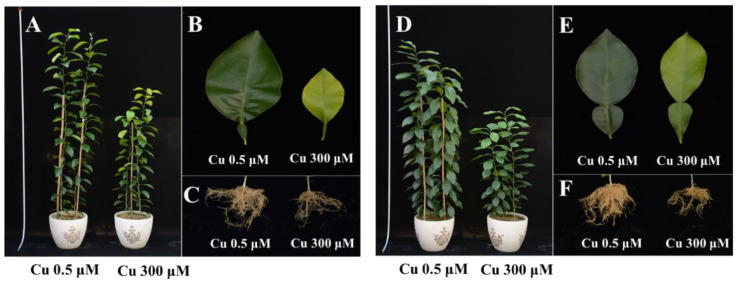
The effects of Cu toxicity on seedling growth (**A**,**D**), leaf morphology (**B**,**E**) and root development (**C**,**F**) of two citrus species (*C. sinensis*: (**A**–**C**); *C. grandis*: (**D**–**F**)).

The citrus seedlings exposed to 300 μM Cu toxicity were significantly lower than the control seedlings in two citrus species (Figure 1A,D). The toxicity of 300 μM Cu decreased the leaf area and caused obvious leaf chlorosis compared with the control of two citrus species (Figure 1B,E). Moreover, the yellowish leaves were observed mainly on the upper leaves compared with the lower leaves under 300 μM Cu in both citrus species. The fresh roots of control seedlings were bright and vigorous. However, citrus roots that suffered from Cu toxicity were dark, with fewer root branches. Remarkably, the root development was inhibited by 300 μM Cu toxicity of two citrus species (Figure 1C,F). 

### 2.2. Cu Toxicity Altered the Biomass Distribution, Decreased the Cu Translocation Factor and Total Chlorophyll Content of C. sinensis and C. grandis

As shown in Table 1, 300 μM Cu toxicity decreased the ratio (shoot biomass/dry weight) by 7.42% and 4.12% in *C. sinensis* and *C. grandis* compared with the control, respectively. Contrastingly, the ratio (root biomass/dry weight) increased by 40.86% and 27.45% under 300 μM Cu toxicity in *C. sinensis* and *C. grandis* compared with the control. The comparison between two citrus species indicated that *C. sinensis* had a significantly lower ratio of shoot biomass to dry weight but a significantly higher ratio of root biomass to dry weight than *C. grandis,* both under control and 300 μM Cu toxicity. Moreover, the control seedlings of *C. grandis* had a noticeably higher Cu translocation from the lateral roots to the leaves than *C. sinensis*. However, the Cu translocation factors of two citrus species decreased significantly under Cu toxicity compared with the control. Under Cu toxicity, no significant difference was found between the two citrus species. Likewise, the total chlorophyll content of the two citrus species was decreased remarkably by Cu toxicity compared with the control without a significant difference between the two citrus species under Cu toxicity. 

### 2.3. Cu Toxicity Disrupted the Anatomical Structure of Leaves and Roots of C. sinensis and C. grandis

**Figure 2:**
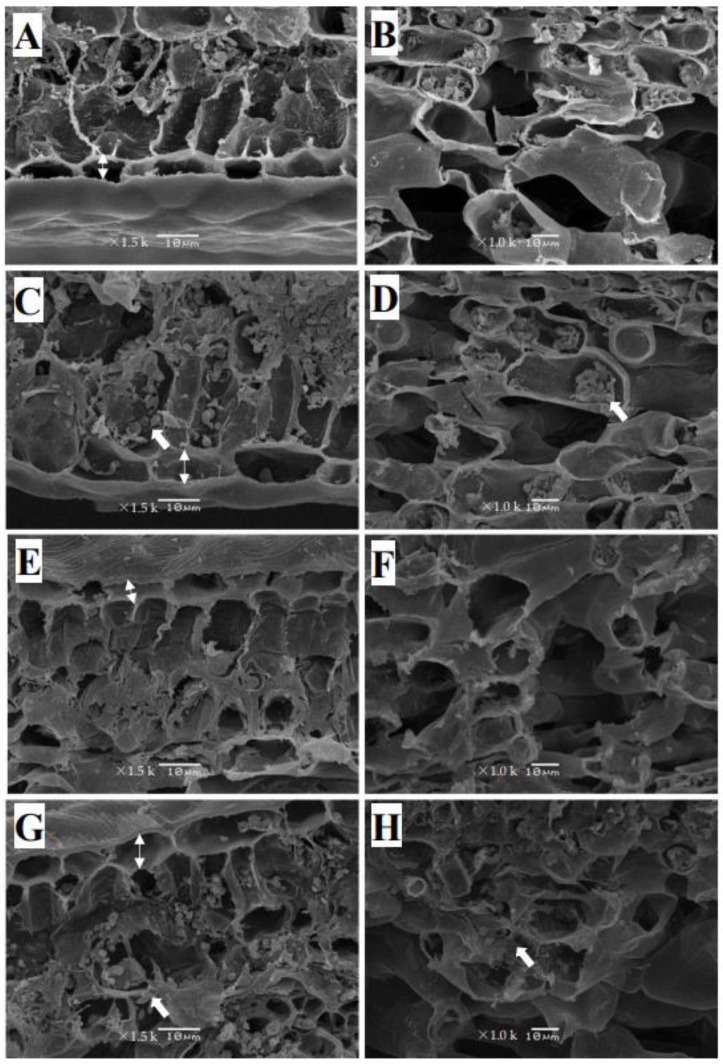
The SEM images of 0.5 µM (**A**,**B**,**E**,**F**) and 300 µM Cu-treated (**C**,**D**,**G**,**H**) leaves of two citrus species. *C. sinensis*: (**A**–**D**); *C. grandis*: (**E**–**H**). Images (**A**,**C**,**E**,**G**) show epidermis and palisade tissues of citrus leaves. Images (**B**,**D**,**F**,**H**) show the spongy tissue. The granules in cells labeled by arrows are starch granules. The two-way arrow represents the average thickness of the leaf epidermis. The scales in the figures represent 10 μm.

The SEM revealed that the epidermal cells of the leaves of *C. sinensis* from the control group were well-structured by a single layer in a rectangular-like shape. The palisade parenchyma could be found under the epidermis, consisting of layers of parenchymal cells in a regular column (Figure 2A). The spongy parenchyma adjacent to the lower leaf epidermis was irregularly distributed, containing a small number of starch granules (Figure 2B). Excessive Cu enlarged the epidermal cells and shrank the palisade parenchyma. Strikingly, the number of starch granules increased in the palisade and spongy parenchyma (Figure 2C,D). Compared with *C. sinensis*, *C. grandis* had a thicker epidermis and shorter palisade parenchyma under control. The number of starch granules in the palisade and spongy parenchyma was less than that in *C. sinensis* (Figure 2E,F). Cu toxicity disrupted the arrangement of spongy parenchyma and significantly increased the number of starch granules in the palisade and spongy parenchyma of *C. grandis* leaves (Figure 2G,H).

**Figure 3:**
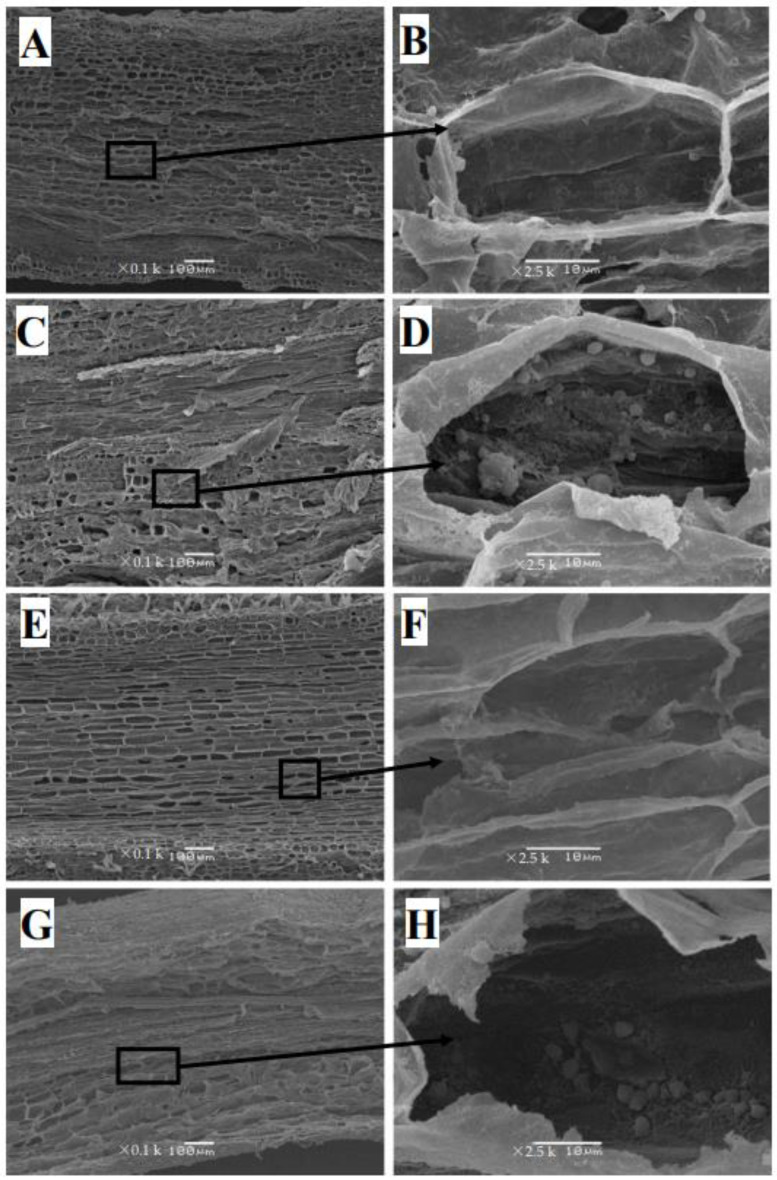
The SEM images of the longitudinal section of 0.5 µM (**A**,**B**,**E**,**F**) and 300 µM Cu-treated (**C**,**D**,**G**,**H**) roots of two citrus species. *C. sinensis*: (**A**–**D**); *C. grandis*: (**E**–**H**). The right figures are partial enlargements of the left figures. The granules in cells labeled by arrows are starch granules. The scales in figures (**A**,**C**,**E**,**G**) represent 100 μm. The scales in figures (**B**,**D**,**F**,**H**) represent 10 μm.

**Figure 4:**
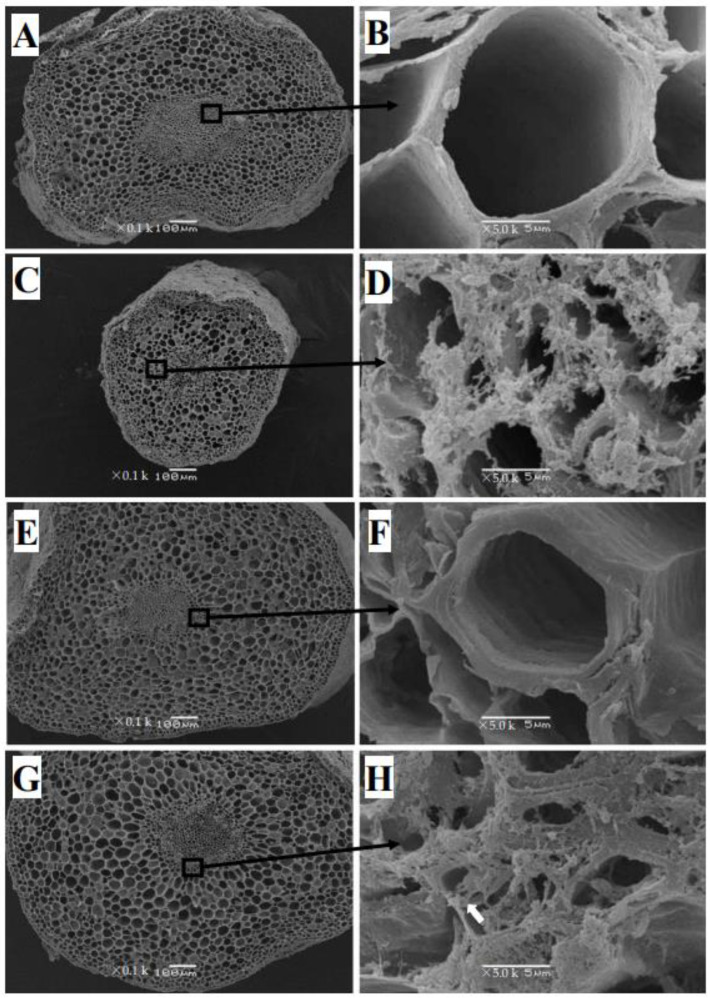
The SEM images of cross-section of 0.5 µM (**A**,**B**,**E**,**F**) and 300 µM Cu-treated (**C**,**D**,**G**,**H**) roots of two citrus species. *C. sinensis*: (**A**–**D**); *C. grandis*: (**E**–**H**). The right figures are partial enlargements of the left figures. Images (**A**,**C**,**E**,**G**) show epidermis, exodermis, cortex, endodermis, pericycle, xylem and phloem. Images (**B**,**D**,**F**,**H**) show the xylem of the roots. The arrows represent the blocked xylem of roots by flocculent precipitate in (**D**) and (**H**). The scales in figures (**A**,**C**,**E**,**G**) represent 100 μm. The scales in figures (**B**,**D**,**F**,**H**) represent 5 μm.

The cell layer in the root exodermis of control *C. sinensis* was well-structured in the transversal and longitudinal sections under SEM (Figure 3A and Figure 4A). The distorted vessels and impaired structure at the cross-section of *C. sinensis* roots were found under Cu toxicity (Figure 3C). The xylem of control *C. sinensis* was almost empty (Figure 3B and Figure 4B). However, the cross-section of the root xylem was covered by flocculent precipitate under Cu toxicity in *C. sinensis* (Figure 4D). Increased accumulation of starch granules was observed in roots of excessive Cu-treated *C. sinensis* (Figure 3D). Compared with *C. sinenis* under control, *C. grandis* had a thicker xylem and the inner ring was clear (Figure 4E,F). The accumulated starch granules, disrupted vessels and flocculent precipitate at the longitudinal section were also found to be in excess of Cu-treated *C. grandis* (Figure 3H and Figure 4H). Compared with *C. sinensis* under Cu toxicity, *C. grandis* had a more apparent flocculent precipitate on the cross sections under SEM. 

### 2.4. Cu Toxicity Destroyed the Ultrastructure of the Leaves and Roots of C. sinensis and C. grandis

**Figure 5:**
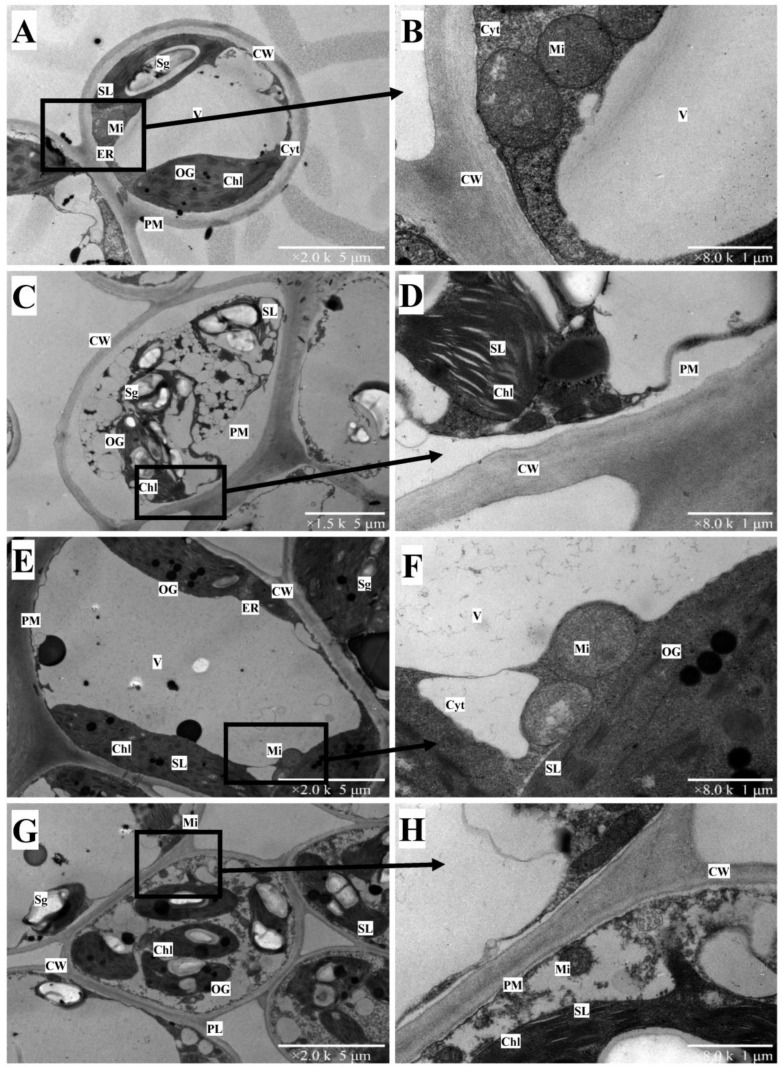
The TEM images of 0.5 µM (**A**,**B**,**E**,**F**) and 300 µM Cu-treated (**C**,**D**,**G**,**H**) leaves of two citrus species. *C. sinensis*: (**A**–**D**); *C. grandis*: (**E**–**H**). The right figures are partial enlargements of the left figures. Chl: chloroplast; CW: cell wall; Cyt: cytoplasm; ER: endoplasmic reticulum; Mi: mitochondrion; OG: osmiophilic globule; PM: plasmalemma; Sg: starch granules; SL: stroma lamella; V: vacuole. The scales in figures (**A**,**C**,**E**,**G**) represent 5 μm. The scales in figures (**B**,**D**,**F**,**H**) represent 1 μm.

Ultrastructural observation under TEM revealed an integral cellular structure of *C. sinensis* leaf under control. As found in Figure 5A, the control leaf cell had an approximately round shape and the membrane structure was clearly visible. The organelles were pushed by a center-located vacuole and closely distributed at the inner side of the plasma membrane. The thylakoids were stacked closely and the lamellar structure was observed inside the chloroplast. Moreover, several starch granules and osmiophilic globules were occasionally found in the chloroplast. The bilayer structure and inner cristae structure of mitochondria were also clearly visible under control (Figure 5B) as was excessive Cu-induced plasmolysis of *C. sinensis* leaf cells (Figure 5C). Moreover, the cellular structure was impaired. The vacuole was broken and replaced by many spherical vesicles. The size of mitochondria decreased, and the bilayer structure was difficult to find under Cu toxicity. The integrity of the chloroplast was destroyed, and the stacked thylakoids were loose (Figure 5D). Strikingly, Cu toxicity increased the number and volume of starch granules in *C. sinensis* leaves.

The control leaf cells of *C. grandis* were presented in an oval-like shape (Figure 5E). The organelles were well-organized. The stroma lamella of chloroplast and bilayer structure of the mitochondria were apparent in the enlargement in Figure 5F. Under Cu toxicity, the vacuole disappeared. The chloroplasts occupied most of the protoplast. The enlarged starch granules could be found in each chloroplast. The mitochondria of *C. grandis* leaf under Cu toxicity were smaller than in the control (Figure 5F). Compared with *C. sinensis*, *C. grandis* had a relatively integral chloroplast structure.

**Figure 6:**
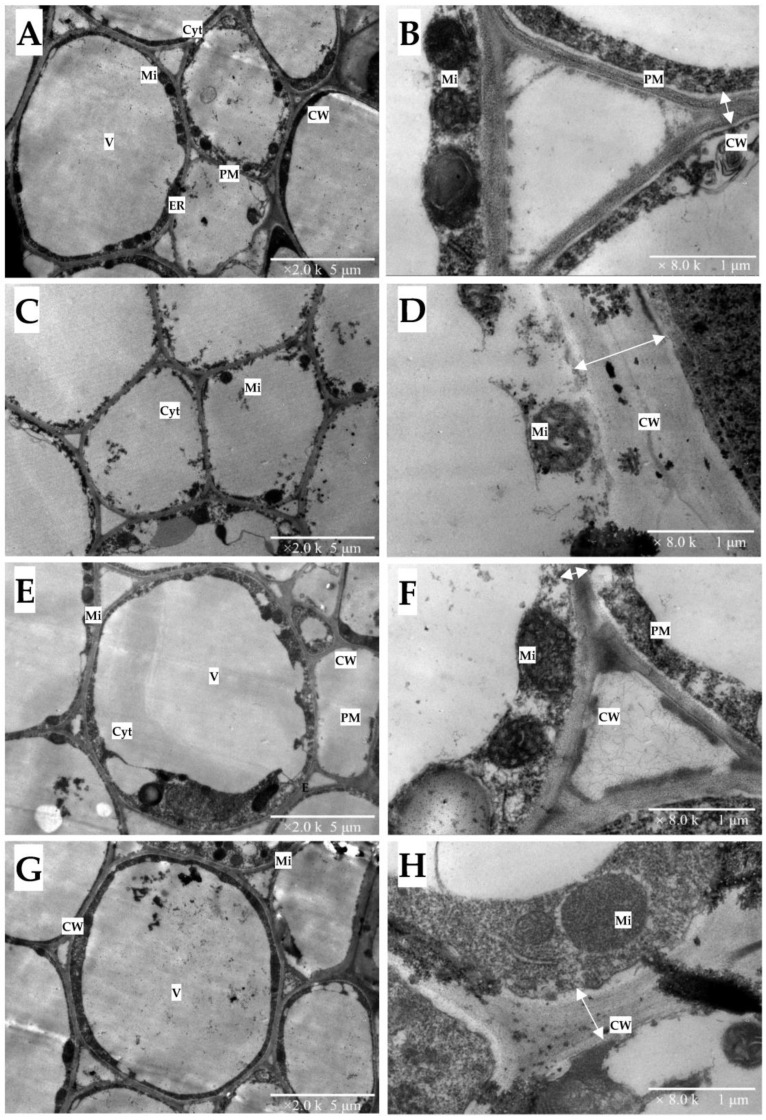
The TEM images of 0.5 µM (**A**,**B**,**E**,**F**) and 300 µM Cu-treated (**C**,**D**,**G**,**H**) roots of two citrus species. *C. sinensis*: (**A**–**D**); *C. grandis*: (**E**–**H**). CW: cell wall; Cyt: cytoplasm; ER: endoplasmic reticulum; Mi: mitochondrion; PL: plasmalemma; V: vacuole. The two-way arrow represents the average thickness of the cell wall. The scales in figures (**A**,**C**,**E**,**G**) represent 5 μm. The scales in figures (**B**,**D**,**F**,**H**) represent 1 μm.

In the root, the vacuole almost took up the protoplast of the control cell in *C. sinensis* (Figure 6A) and *C. grandis* (Figure 6E). The mitochondria were distributed on the plasma membrane’s inner side (Figure 6B,F). Under Cu toxicity, the vacuole disappeared in *C. sinensis* roots. The mitochondrial structure was also difficult to observe (Figure 6C,D). Differentially, *C. grandis* maintained a relatively integral structure of the root cell, supported by an intact structure of mitochondria and a clear edge between the plasma membrane and cell wall (Figure 6G). Interestingly, both citrus cell wall thicknesses increased under Cu toxicity compared with the control.

## 3. Discussion

Heavy metal (HM) stress had detrimental effects on seed germination and membrane stability of horticultural plants, which inhibited plant growth and crop yield [25]. Similarly, Cu toxicity decreased the leaf chlorophyll content and depressed the shoot and the root development of citrus species, which is in line with our previous reports [26,27]. Additionally, Cu toxicity significantly downregulated the shoot biomass to dry weight ratio whereas it upregulated the root biomass to dry weight of the two citrus species (Table 1). The changes in biomass distribution might be due, at least in part, to the limited nutrient transport to the shoots. The Cu-toxicity-induced increased ratio of root biomass to shoot biomass agreed with that in excessive-Cu treated *Hymenaea courbaril* L. (Caesalpinioideae), a promising woody species for phytoremediation [28]. Cu toxicity significantly decreased the total chlorophyll content of the two citrus species (Table 1). The decrement was also reported in Cu-stressed falx leaves [29]. Coherently, the total chlorophyll content of soybean leaf decreased with the increasing level of soil Cu [30].

Cu immobilized by the roots contributed to Cu detoxification of horticultural plants [31]. The present results indicate that *C. grandis* had a significantly higher TF than *C. sinensis* under control. However, no significant difference in TF was found between the two citrus species under Cu toxicity (Table 1). This finding suggests that a potential strategy governing Cu mobility might exist in *C. grandis* under Cu toxicity. This hypothesis was evidenced by the upregulation of genes involved in Cu-binding in excessive-Cu-treated roots of *C. grandis* [20]. Beyond the transcriptional evidence, it has also been reported that tiny crystals accumulated in the root xylem of Cu-stressed Moso Bamboo (*Phyllostachys pubescens*), which block the Cu transport in the vascular tissue of the root xylem [32]. In the present study, citrus roots that received Cu excess had similar amorphous material accumulated on the xylem (Figure 4D,H). Interestingly, much more flocculent precipitate was found in *C. grandis* than in *C. sinensis*.

The anatomical investigation provided essential information for understanding the Cu-adaptation of citrus species. Herein, we found Cu toxicity increased starch granules accumulation in the leaf palisade and spongy parenchymatous tissues (Figure 2C,D,G,H) and root cells (Figure 3D,H) of two citrus species compared with control. Li et al. [26] demonstrated the increased starch accumulation in the leaves and roots of two citrus species. It has been proposed that Cu-toxicity-induced starch accumulation in cucumber leaves is related to a decreased phloem loading [33], leading to feedback inhibition of photosynthesis. Meanwhile, the accumulated starch in the root has been considered an osmotic regulation in turgor pressure under stress [34,35]. Those findings, and an increased ratio of root biomass to plant dry weight, imply a crucial role of starch accumulation in citrus roots conferring Cu toxicity. Likewise, the enhanced starch content has also been reported in excessive Cd-treated poplar species [36] and apple seedlings [37]. Additionally, Cu toxicity enhanced the thickness of the leaf epidermis of two citrus species compared with the control (Figure 2C,G). The leaf thickening has also been observed in Cu-stressed oregano leaves [38] and Al-treated sunflower (*Helianthus annuus* L.) leaf [39]. Bouazizi et al. [40] have proposed that the Cu-toxicity-induced cell wall lignification contributes to cell wall thickening, which ultimately strengthens the Cu tolerance of bean leaves. Further studies have focused on phenylalanine ammonia-lyase activity (PAL, EC 4.3.1.5), a rate-limited enzyme in lignin synthesis, and research on the lignin deposited on the cell wall should be carried out to disclose the structural modification of Cu toxicity on citrus leaves and roots.

Based on TEM observation, we believe that the ultrastructural alterations reflect the status of plant organelles under heavy metal toxicity, which are complementary to the plant’s physiological parameters [41]. For instance, our previous report has proven that Cu toxicity resulted in oxidative damage of citrus leaves by overproduction of reactive oxygen species [19]. It has also been further reported that oxidative stress accounted for the membrane damage of plant cells exposed to excess Cu [42]. Therefore, the present results, in which Cu-toxicity induced damage to the membrane structure of citrus leaves, verify our previous findings and extend our knowledge of the response to Cu toxicity in citrus roots and leaves. Additionally, it was striking to find a severely disrupted structure of chloroplast and increased starch granules in Cu-stressed *C. sinensis* leaves (Figure 5D). Studies have proposed that heavy metal stress decreased the leaf chlorophyll content by downregulating the activities of enzymes involved in chlorophyll synthesis [43,44]. Similarly, the inhibited alpha-amylase and invertase isoenzymes have been reported for their restricted breakdown of starch granules, leading to starch accumulation under Cu toxicity [45]. The increasing number of starch granules has also been found in excessive-Mn-treated leaves of *Xanthium strumarium* L., a candidate species for Mn-phytoremediation [46]. Compared with Cu-treated *C. sinensis* roots, *C. grandis* had a relatively robust chloroplast structure (Figure 5C,G).

The thickening of the cell wall is significant for plant roots conferring heavy metal toxicity [47]. Primarily composed of polysaccharides (such as pectin, cellulose and lignin), the cell wall had numerous negatively charged groups, such as hydroxyl (–OH^−^) and carboxyl (–COOH^−^) groups. Thickening the cell wall increased the negative charge, which restricted the translocation of the toxic cation. In the present study, the observation under stereoscopic microscopy verified that Cu toxicity expanded the fibrous roots of two citrus species (Figure S1). Furthermore, the TEM observation of citrus roots indicated that the root cell wall’s increased thickness might account for the root tips’ expansion (Figure 6D,H). Our previous finding that Cu toxicity increased the ratio of cell wall biomass to the dry weight of citrus roots is in line with present results [20]. Similar findings have also been reported in excessive-Cu treated roots of *Pinus pinaster* Ait (Arduini et al., 1995), *Arabidopsis thaliana* [48] and *Allium cepa* L. [49]. The comparison between Figure 6C,G implies that *C. grandis* maintained a much more integral vacuole structure.

## 4. Materials and Methods

### 4.1. Plant Material and Treatments

Surface-sterilized seeds of *C. sinensis* and *C. grandis* were germinated in moist sand in a greenhouse of Fujian Agriculture and Forestry University, Jinshan campus (26°5′ N, 119°14′ E) in March 2020. Six weeks after germination, the uniformly sized seedlings (about 10 cm) were selected and precultured with 1/4 strength modified Hoagland nutrient solution, which contained 2.5 mM KNO_3_, 2.5 mM Ca(NO_3_)_2_, 0.5 mM KH_2_PO_4_, 1 mM MgSO_4_, 10 µM H_3_BO_3_, 2 µM MnCl_2_, 2 µM ZnSO_4_, 0.5 µM CuCl_2_, 0.065 µM(NH_4_)_6_Mo_7_O_24_ and 20 µM Fe-EDTA. Six weeks after preculture, the citrus seedlings were treated with nutrient solution (pH = 4.30–4.50) containing 0.5 µM CuCl_2_ (as control) or 300 µM CuCl_2_ (as Cu toxicity) daily for 18 weeks. By the end of the treatments, citrus leaves, stems and roots were sampled separately. For sampling, the citrus roots were first washed with tap water to remove excess sandy particles, followed by soaking in 0.5 M EDTA-Na_2_ for 10 min (three times) to remove Cu residue on the root surface. After being washed with distilled water three times, fresh lateral roots of citrus seedlings were fixed in 3% glutaraldehyde–1.5% paraformaldehyde solution (fixing solution) in 0.1 M phosphate buffer solution (PBS, pH = 7.2) to prepare the sample for microscopic observation. There were three replicates for each treatment. After being cleaned with distilled water, the remaining fresh citrus leaves were collected for chlorophyll content measurement. The remaining tissues of citrus seedlings were dried before biomass analyses and Cu quantification. There were four replicates of each treatment for biomass analyses.

### 4.2. Measurements of Cu Translocation Factor and Leaf Total Chlorophyll Contents of Two Citrus Species

The Cu concentration of plant tissues and the leaf chlorophyll content were measured according to Li et al. [16]. The Cu translocation factor was expressed as the ratio of Cu concentration in the leaves to the lateral roots [50]. The leaf total chlorophyll content was calculated as: 7.15 × A_663_ + 18.71 × A_646_, in which, A_663_ and A_646_ represented the absorbances of leaf pigment extractant under the wavelength of 663 and 646, respectively. There were four replicates of each treatment.

### 4.3. Scanning Electron Microscopy (SEM) and Transmission Electron Microscopy (TEM) Analyses of Citrus Leaves and Roots

The pretreatment of citrus leaves and roots was performed according to Huang et al. [51]. Briefly, the citrus leaf and root samples were vacuumed in a syringe filled with fixing solution under 4 °C for 3 h, followed by three rinses in PBS, each lasting 15 min. The samples were then post-fixed with 1% OsO_4_–1.5% potassium hexacyanoferrate for 2 h and washed with distilled water three times. Thereafter, the citrus leaf and root samples were dehydrated in an increasing concentration of ethanol (30%, 50%, 70%, 80%, 90%, 95% and 100%) three times.

SEM observation: The dehydrated samples were rinsed in epoxypropane twice and dried by an HCP-2 critical point dryer (HITACHI Ltd., Tokyo, Japan). Further, the samples were mounted on brass stubs coated with gold in Eiko IB-5 ion-coater (Eiko Engineering, Ibaragi, Japan). The images of citrus tissues were obtained using a JEOL JSM-6380LV electron microscope (JEOL, Ltd., Tokyo, Japan). There were three replicates of each treatment.

TEM observation: The dehydrated samples were rinsed with acetone for 10 min twice, and then replaced by a mixture of resin and acetone on a shaker at the ratios of 1:1 and 3:1 for 2 h, respectively. The samples were then dried with filter paper to remove excess acetone on the surface and infiltrated in resin overnight. After being embedded in epoxy resin 618, the individual leaf and root blocks were cut into sections of approximately 80-nm thickness with an ultra-microtome LEICA EM UC6 (Leica, Wetzlar, Germany). The sections were stained with 2% uranyl acetate before observations with a TEM HITACHI HT7700 (HITACHI, Tokyo, Japan) equipped with a digital camera. There were three replicates of each treatment.

### 4.4. Statistical Analysis

The data in the study represent mean ± SE (*n* = 4). Four means (two citrus species × two treatments) were analyzed by two ANOVAs followed by Duncan’s multiple range tests at *p* < 0.05 using SPSS (Version 22.0, IBM, Armonk, NY, USA).

## 5. Conclusions

Cu toxicity induced by 300 μM Cu significantly decreased the ratio of shoot biomass to dry weight, the Cu translocation factor and the total chlorophyll of two citrus species. By contrast, the ultrastructural alterations demonstrated that the starch granules in the leaves and roots, the root flocculent precipitate and the thickness of the root cell wall all increased under Cu stress in the two citrus species. The increased root flocculent precipitate and thickened root cell wall might reduce the Cu translocation from roots to the shoots of the two citrus species. Compared with *C. sinensis, C. grandis* maintained a relatively integral root cellular structure under Cu toxicity, which provided a structural basis for a higher Cu tolerance than *C. sinensis*. The present results increase our understanding of the morphological alteration of citrus leaves and roots under Cu toxicity. Further study regarding root metabolites under Cu toxicity should be undertaken to reveal the Cu-tolerant mechanisms of citrus species underlying the structural alteration.

## Figures and Tables

**Table 1:** The effects of excessive Cu on biomass distribution, Cu translocation factor (TF, defined as the ratio of Cu concentration in the leaves to the lateral roots) and total chlorophyll content of two citrus species. The values represent means *±* SE of four replicates. Significant differences (*p* ≤ 0.05) between treatments are indicated by different letters.

Species	Treatments	Shoot Biomass/Dry Weight (%)	Root Biomass/Dry Weight (%)	Cu TF (%)	Total Chlorophyll (μg/cm^2^)
*C. sinensis*	Cu 0.5 μM	84.63 ± 0.26 b	15.37 ± 0.26 b	27.08 ± 0.08 b	73.22 ± 1.27 a
	Cu 300 μM	78.35 ± 0.90 c	21.65 ± 0.90 a	0.53 ± 0.04 c	66.16 ± 1.09 bc
*C. grandis*	Cu 0.5 μM	86.97 ± 0.85 a	13.03 ± 0.85 c	40.46 ± 3.65 a	68.73 ± 2.82 ab
	Cu 300 μM	83.39 ± 0.83 b	16.61 ± 0.83 b	0.52 ± 0.06 c	61.59 ± 2.89 c

## Data Availability

Data are archived in L.-S. Chen’s lab and are available upon request.

## Supplementary Materials

The following supporting information can be downloaded at: https://www.mdpi.com/article/10.3390/plants12020351/s1, Figure S1: The root tip morphology of two citrus species under 0.5 µM and 300 µM Cu stress. The image was taken with stereoscopic microscopy SMZ18 (Nikon, Tokyo, Japan).
