# Supplementary material for: Physiological and Ultrastructural Responses to Excessive-Copper-Induced Toxicity in Two Differentially Copper Tolerant Citrus Species
Source: Plants (Basel). 2023 Jan 11;12(2):351. doi: 10.3390/plants12020351 (PMC9866615; doi:10.3390/plants12020351)
Supplement: Supplementary file 1 [file plants-12-00351-s001.zip › plants-2144099-supplementary.pdf]

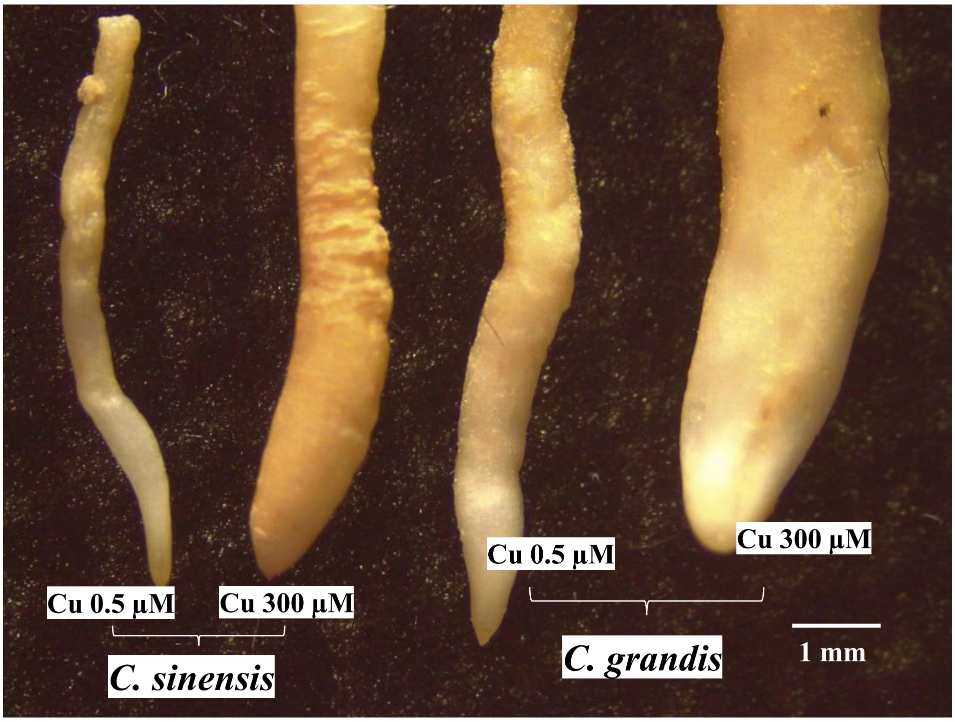

Figure S1: The root tip morphology of two citrus species under 0.5 μM and 300 μM Cu stress. The image was taken with stereoscopic microscopy SMZ18 (Nikon, Tokyo , Japan)
